# The Crystal Structure of *Bacillus thuringiensis* Tpp80Aa1 and Its Interaction with Galactose-Containing Glycolipids

**DOI:** 10.3390/toxins14120863

**Published:** 2022-12-08

**Authors:** Hannah L. Best, Lainey J. Williamson, Magdalena Lipka-Lloyd, Helen Waller-Evans, Emyr Lloyd-Evans, Pierre J. Rizkallah, Colin Berry

**Affiliations:** 1 School of Biosciences, Cardiff University, Park Place, Cardiff CF10 3AX, UK; 2 School of Pharmacy, Cardiff University, Park Place, Cardiff CF10 3AX, UK; 3 School of Medicine, Cardiff University, Heath Campus, Cardiff CF14 3XN, UK

**Keywords:** *Bacillus thuringiensis*, Tpp80Aa1 toxin, crystal structure, *Culex*, *Anopheles*, *Aedes*, biocontrol, pesticidal protein

## Abstract

Tpp80Aa1 from *Bacillus thuringiensis* is a Toxin_10 family protein (Tpp) with reported action against *Culex* mosquitoes. Here, we demonstrate an expanded target range, showing Tpp80Aa1 is also active against the larvae of *Anopheles gambiae* and *Aedes aegypti* mosquitoes. We report the first crystal structure of Tpp80Aa1 at a resolution of 1.8 Å, which shows Tpp80Aa1 consists of two domains: an N-terminal β-trefoil domain resembling a ricin B lectin and a C-terminal putative pore-forming domain sharing structural similarity with the aerolysin family. Similar to other Tpp family members, we observe Tpp80Aa1 binds to the mosquito midgut, specifically the posterior midgut and the gastric caecum. We also identify that Tpp80Aa1 can interact with galactose-containing glycolipids and galactose, and this interaction is critical for exerting full insecticidal action against mosquito target cell lines.

## 1. Introduction

The *Bacillus thuringiensis* (Bt) is a Gram-positive, sporulating bacterium that can produce a range of insecticidal toxins that are active across an array of invertebrate targets [1]. Bt-produced proteins show highly selective and potent activity and, as such, have revolutionised agriculture by negating the use of hazardous and non-specific chemical pesticides. Additionally, Bt proteins are used for the control of insect vectors of human disease-principally mosquitoes [2]. The importance and commercial success of Bt proteins motivates ongoing searches for novel proteins with new spectra of insecticidal activity.

Tpp80Aa1 (formerly Cry80Aa1) is a recently identified mosquitocidal protein, discovered by whole genome sequencing of Bt strain S3589-1 isolated from a soil sample [3], and it is reported to be active against third instar *Culex pipiens pallens* larvae. Control of disease-spreading Diptera—such as mosquitoes and black-fly—is critical for the control of diseases such as Zika virus, malaria, dengue, yellow fever, and African river blindness [4,5]. To date, the most successful entomopathogenic bacteria for controlling these populations in the field have been Bt serovar. *israelensis* (Bti) and *Lysinibacillus sphaericus.* Bti produces several Cry and Cyt toxins in the form of crystalline inclusions, which allow the bacterium to demonstrate significantly stronger toxicity due to the synergism between individual proteins within the whole Bti crystal [6]. The most utilised *L. sphaericus* toxins are a heterodimer of Tpp1Aa1 and Tpp2Aa1 (formerly BinA and BinB), which are highly potent against *Culex* and *Anopheles* larvae [7], and can also act synergistically with Cyt1Aa to become active against *Ae. aegypti* and Tpp1/2-resistant larvae [8,9,10]. Tpp1Aa1/Tpp2Aa1-producing *L. sphaericus* strains are heavily utilised in mosquito control in the field, and the emergence of resistance incentivises the discovery of new mosquitocidal proteins—such as Tpp80Aa1—that can be used as stand-alone agents, or in a synergistic approach [11,12].

Bacterial pesticidal proteins belong to one of several distinct structural classes [13]. Tpp proteins have two domains, an N-terminal trefoil domain and a C-terminal pore-formation domain [14]. Conserved domain analysis of Tpp80 indicated the greatest identity to Tpp78Aa1 (38.6%)—a protein with activity against the Hemiptera *Nilaparvata lugens* and *Laodelphax striatellus* and the Tpp78Aa1 structure has recently been published [15]. In this study we report the structure of Tpp80Aa1 (1.8 Å), which consists of an N-terminal ricin B lectin domain and a C-terminal toxin_10 putative pore-forming domain (PFD). We demonstrate that the protein has an affinity for galactose-containing glycans, which may mediate its activity. We also reveal an expanded range of activity against new target mosquito species, *An. gambiae* and *Ae. aegypti*, increasing the protein’s value as a novel toxin for mosquito control.

## 2. Results

### 2.1. Tpp80Aa1 Has Activity against C. quinquefasciatus, Ae. aegypti, and An. gambiae Larvae and Mosquito Cell Lines

**Figure 1:**
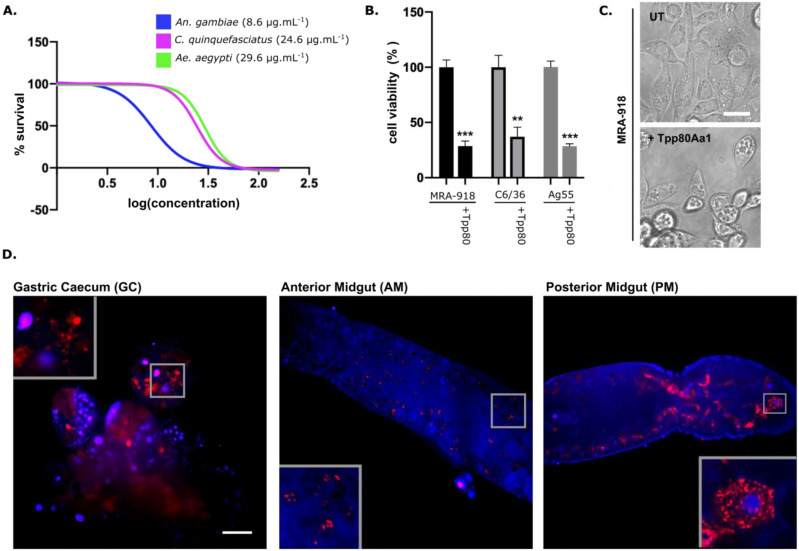
Tpp80Aa1 is active against *Culex quinquefasciatus*, *Aedes aegypti*, and *Anopheles gambiae*. (**A**) Dose response curve of Tpp80Aa1 added to larvae in water plotting log concentration (μg/ mL) against % of surviving larvae. The 50% lethal concentrations (LC_50_) were determined as 8.6, 24.6, and 29.6 μg/mL for *An. gambiae, C. quinquefasciatus,* and *Ae. aegypti*, respectively. (**B**) Cell lines isolated from *C. quinquefasciatus* (MRA-918), *Ae. aegypti* (C6/36), and *An. gambiae* (Ag55) show significantly reduced viability (as determined via resazurin assay) 48 h post addition of Tpp80Aa1 at 50 μg/mL. Data are presented as % of control ± SD and were analysed using unpaired *t*-tests (*** *p* ≤ 0.0001, ** *p* = 0.0014) (**C**) Light microscopy images of untreated (UT) or Tpp80Aa1 treated *C. quinquefasciatus* cells (MRA-918), 48 h post addition. Representative scale bar in the UT image = 10 μm. (**D**) Tpp80Aa1 fluorescently labelled with Alexa Fluor^®^ 555 (red) was fed to *Ae. aegypti* larvae via addition to water. Post-ingestion, larvae were transferred to fresh water for 30 min before guts were dissected, labelled with Hoecsht 33342 (blue), and imaged with a single plan illumination microscope. Grey insets showing punctate binding pattern of Tpp80Aa1. Representative scale bar in GC image = 100 μm.

In mosquitocidal bioassays, trypsin-activated Tpp80Aa1 (Figure S1A,B) demonstrated activity against *An. gambiae, C. quinquefasciatus*, and *Ae. aegypti* larvae, with LC_50_ values of 8.6, 24.6 and 29.6 μg/mL, respectively (Figure 1A). Only *Culex pipiens pallens* (LC_50_ 72 μg/mL) susceptibility has been reported previously [3]. Compared to other mosquitocidal Tpp proteins, Tpp80Aa1 has a lower potency but a broader target range of activity (Table 1). Cellular models were used to investigate Tpp80Aa1 toxicity in vitro. In line with the larval bioassays, 48 h after the addition of Tpp80Aa1, cell viability was substantially reduced in MRA-918 (*C. quinquefasciatus*), C6/36 (*Ae. aegypti*), and Ag55 (*An. gambiae*) cell lines (Figure 1B). Inspection of cellular morphology, via light microscopy, at the 48 h time point shows cells exposed to Tpp80Aa1 have a rounded morphology and are becoming detached from the plate—indicative of cell death. Vacuolisation is also present in the Tpp80Aa1-treated cells (Figure 1C), a cellular phenotype that has been described previously with other Tpp family member two-component toxins, Cry48/Tpp49 [16] and Tpp1/Tpp2 [17,18,19]. This establishes these cell lines as models for further investigations into the Tpp80Aa1 mechanism of action.

### 2.2. Tpp80Aa1 Binding Occurs Predominantly in the Posterior Midgut

To investigate the gut binding profile of Tpp80Aa1, we fed fluorescently labelled Tpp80Aa1 to *Ae. aegypti* larvae (Figure 1D). Tpp80Aa1 binding is predominantly present in the posterior midgut (PM) and gastric caecum (GC), with substantially weaker binding observable in the anterior midgut (AM). A clear punctate staining pattern is present throughout the midgut—particularly in the PM—suggesting internalisation of Tpp80Aa1 in cytoplasmic vesicles. This binding pattern is very similar to that previously reported with radio-labelled or fluorescently labelled Tpp1/Tpp2 in the *Culex* midgut [7,20,21] and may suggest that the elusive Tpp80Aa1 receptor(s) is specifically localised to brush border membranes of the PM and GC. The localisation pattern of the binding may also be due to the known pH gradient throughout the mosquito midgut (approx. pH 8 in the gastric caecum, >pH 10 in the anterior midgut, falling to pH 7.5 in posterior midgut) [22,23] affecting the processing and binding of Tpp80Aa1.

**Table 1:** Summary of LC_50_ values (μg/mL) of mosquitocidal Tpp proteins.

	*Anopheles gambiae*	*Culex quinquefasciatus*	*Aedes aegypti*
Tpp80Aa1	8.6	24.6	29.6
Cry48/Tpp49	NT *	0.02/0.006 [24]	NT *
Tpp1/Tpp2	0.013–0.03 [20]	0.013–0.03 [24]	No toxicity to very low toxicity–depending on variant [25,26]

* NT = reported nontoxic.

### 2.3. Tpp80Aa1 Structure Description

**Figure 2:**
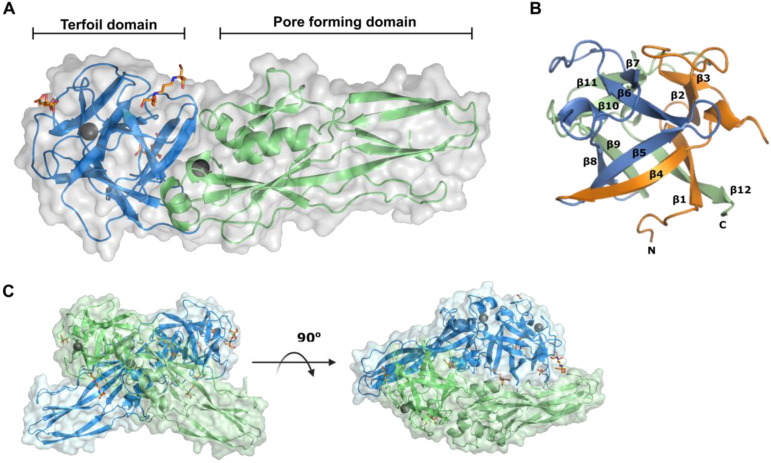
Tpp80Aa1 structure and homodimer packing. (**A**) Cartoon representation of Tpp80Aa1 shows an N-terminal Ricin-B like-lectin domain (blue) and a C-terminal putative pore-forming domain (green). Two bound calcium ions are represented by the black spheres and orange sticks represent buffer molecules (2 bis-tris propane, 1 citrate). (**B**) The carbohydrate binding domain is composed of three pseudo symmetric sections of β-trefoil fold corresponding with residues 9–57 (orange), 58–105 (blue), and 106–155 (green). (**C**) Tpp80Aa1 is present as a homodimer with a large molecular interface between monomer 1 (blue) and monomer 2 (green).

Our final model had an R_work_/R_free_ of 0.17/0.21 at 1.8 Å resolution and showed Tpp80Aa1 packs into the crystal lattice as homodimers, which could be indexed in the monoclinic space group C 1 2 1 (Table S1). The electron density map showed continuous density for residues 5–355 of the 373 aa wild-type Tpp80 protein sequence (Figure S2). Within each Tpp80Aa1 monomer, two distinct conserved domains appear: an N-terminal ricin B-lectin domain (IPR035992) spanning residues 5–155, and a C-terminal Toxin_10 domain (IPR008872) spanning residues 156–355 (Figure 2A). In the Tpp80Aa1 homodimer structure we also see the presence of two calcium ions (1 per monomer) and five buffer molecules present in the crystallization solution; four bis-tris propane and one citrate (Figure 2).

**Figure 3:**
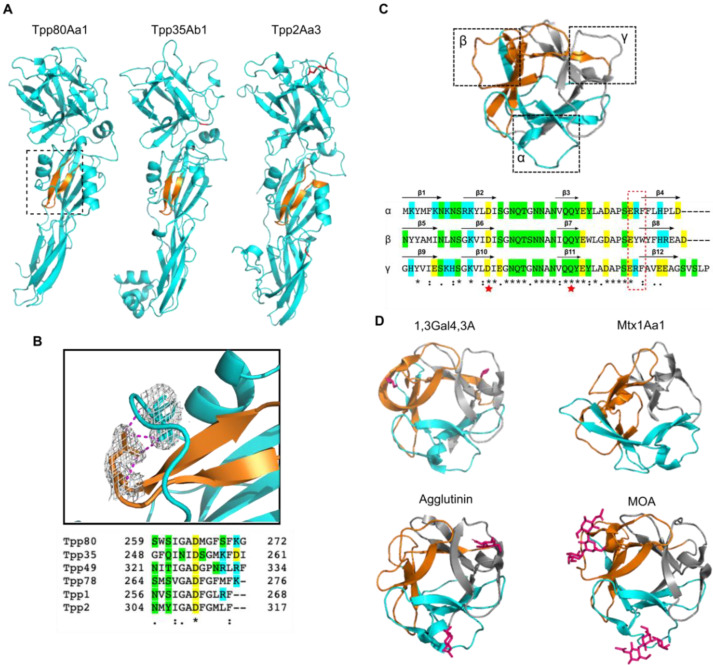
Tpp80Aa1 shows structural homology to other Tpp family members, and ricin B-lectin domains. (**A**) Cartoon depiction of Tpp80Aa1 and insecticidal Tpp family members with strong structural homology, Tpp35Ab1 and Tpp2Aa3. The putative insertion loop is depicted in orange, and disulphide bonds in Tpp35 and Tpp2 are shown in red (there are no Cys residues in Tpp80Aa1). (**B**) Cartoon depiction of the PFD putative insertion loop, boxed in (**A**). The PFD β-hairpin contains a conserved aspartic acid residue that forms polar contacts (magenta) with the backbone of histidine 291 and side chain of serine 290 on the adjacent loop. The conserved aspartic acid residue is marked with an (*) in the sequence alignment of all known Tpp structures, (:) indicates conservation between groups of strongly similar properties, and (.) indicates conservation between groups of weakly similar properties (determined by Clustal Omega). Residues are highlighted cyan = basic amino acids, yellow = acidic amino acids, green = polar uncharged side chains. (**C**) Lectin domain of Tpp80Aa1, highlighting the 3 subdomains α (cyan), β (orange) and γ (grey). A sequence alignment of the 3 domains indicating the regions of repeated β-sheet topology. The region where the ‘QxW’ motif is often found in ricin domains is boxed in red, and the regions of putative carbohydrate-binding residues in related structures are highlighted by a red star. (**D**) Cartoon depiction of lectin domains with strong structural homology include 1,3Gal4,3A bound to glycerol, MOA bound to Gal(1,3)Gal(1,4)GlcNAc, R. solani agglutinin bound to N-acetylgalactosamine, and Mtx1Aa1, with ligands are depicted in magenta. Sequence alignments were generated by Clustal Omega.

The N-terminal domain is composed of the well-described ricin B type β-trefoil lectin fold [27]. The β-trefoil consists of three subdomains α (β1–β4), β (β5–β8), and γ (β9–β12) assembling around a pseudo three-fold axis. The first and fourth β strand of each repeat form together a β-barrel, whereas the second and third form a β-hairpin (Figure 2B and Figure 3C). The C-terminal domain of Tpp80Aa1 is rich in β-sheet topology characteristic of other Tpp proteins (Figure 3A) and the founding member of β-pore-forming toxins (β-PFTs), aerolysin [28]. Aerolysin and aerolysin-like proteins have a structurally conserved PFD, generally consisting of five β-strands with an insertion loop between strands β2 and β3 [29]. A structure reminiscent of an aerolysin insertion loop is present in Tpp80Aa1 between residues 259–272 (SWSIGADMGFS), as a short β-hairpin—with predominantly amphipathic structure—tucked under a loop. Similar structures are present in other Tpp proteins and are hypothesised to unfold in pore formation (Figure 3A and Figure S4).

### 2.4. Interface Analysis

The final model shows the presence of a homodimer with a large molecular interface forming an ‘X’ structure (Figure 2C). Superposition of the Tpp80Aa1 monomers show the two copies to be highly similar with a root-mean-square deviation (RMSD) of 0.795Å estimated by PyMOL [30] (Figure S3A). This ‘X’ structure between Tpp monomers has also been observed in natural crystals of Tpp49Aa1 homodimers [31], and Tpp1Aa2/Tpp2Aa2 heterodimers [32], showing that similar dimer forms are produced in in vitro crystal screens. In the case of Tpp80Aa1, PDBePISA interface analysis identified 12 interfaces (Table S2); the largest interface (1010.9 Å^2^) between the two monomers in the ‘X’ shape involves 34 residues of monomer 1 and 35 residues of monomer 2, with 13 hydrogen bonds (Figure S3B). Although the size of the Tpp80Aa1 molecular interface (1010.9 Å^2^) exceeds the threshold estimated to discriminate between a biological and an artificial dimer (856 Å^2^) [33], it is significantly smaller than that observed for the Tpp1/Tpp2 heterodimer (1833.1 Å^2^) and has a much lower binding energy (Tpp80Aa1 Δ^i^G of −7.1 kcal/mol, Tpp1/Tpp2 of −22.5 kcal/mol). Whereas the large Tpp1/Tpp2 interface may be preserved in solution, this is unlikely to be the case for the Tpp80Aa1 dimer—indeed SEC indicates Tpp80Aa1 is largely monomeric in solution (Figure S1A). Mechanistically this makes sense, given the Tpp1/Tpp2 1:1 molar ratio shown to be optimal for receptor binding and toxicity is 1:1. As Tpp80Aa1 does not need a partner to elicit toxicity, it is possible this ‘X’ shape is a requirement for packing and stability in the crystal structure.

Insecticidal proteins are usually produced in protoxin form and processed, often by trypsin-like enzymes, in the target insect gut [34,35]. N-terminal sequencing by Edman degradation shows the first 5 amino acid residues of trypsin-activated Tpp80Aa1 are XMTFX (where X is an amino acid that could not be assigned). Combined with LCMS analysis, which showed the molecular weight of activated Tpp80Aa1 to be 41,115.5 Da (Figure S1), this indicates that proteolytic activation removes the first three residues and the last eleven amino acids of Tpp80Aa1. In the Tpp1/Tpp2 heterodimer, the large 53-residue Tpp2 pro-region is hypothesised to maintain the heterodimer until receptor binding, where the slow release of the large pro-region signals pore formation [32]. Although the Tpp80Aa1 pro-region fragments are not visible in the electron density map, the pro-regions are located near crystal contact interfaces, and could play a role in stability prior to dissolution (Figure S3B–D).

### 2.5. Tpp80Aa1 Has Structural Similarity with Other Tpp Proteins and Ricin B-like Lectin Domain-Containing Proteins

Tpp80Aa1 structurally related proteins were identified by using the DALI server to search the Protein Data Bank (Figure 3, Table S3). The strongest matches were other Tpp insecticidal proteins: L. sphaericus Tpp2Aa2 (PDB 5FOY-B) and B. thuringiensis Tpp35Ab2 (PDB 4JP0-A). The related Tpp structures all share the N-terminal β-trefoil and C-terminal Toxin_10 family PFD (Figure 3A), and, within the PFD, a putative membrane insertion β-hairpin tucked under a loop. Multiple sequence alignment of the β-hairpin from all published Tpp structures indicated a conserved aspartic acid residue (Figure 3B), which forms polar contacts with the backbone of a residue in the overlying loop, and frequently with the sidechain of a semi-conserved serine/threonine residue preceding it in the loop (Figure 3B and Figure S4).

In addition to the structural homology with other Tpp proteins, Tpp80Aa1 shows strong regional matches with the mosquitocidal holotoxin (Mtx1Aa1, PDB 2VSE-A), which belongs to a distinct structural class of toxin but contains 4 ricin-lectin repeats; an exo-beta-1,3-galactanase (Ct1,3Gal43A; PDB 3VSF-F) from the thermophilic bacterium *Clostridium thermocellum*; and agglutinins from both the fungi *Marasmius oreades* (MOA; PDB 5D63-A), and *Rhizoctonia solani* (PDB 4G9N). These homology matches are based on the structural similarity of the N-terminal ricin_B lectin-like domain (Figure 3D). Ricin B-lectins are frequently characterised by galactose binding, and this has been shown for the four lectin-domains with the greatest structural similarity. Mtx1Aa1 is a mosquitocidal protein comprising a catalytic domain with 4 ricin B-type lectin domains curled around it, containing 12 putative sugar binding sites [36]. These sites are structurally related to pierisin—a cytotoxin from *Pieris rapae* (cabbage white butterfly) that is reported to bind the glycolipids globotriaosylceramide (Gb3) and globotetraosylceramide (Gb4) which have a terminally linked galactose and N-acetylgalactosamine, respectively [37]. Furthermore, the structurally homologous proteins noted above—MOA [38], Ct1,3Gal43A protein [39], and *R. solani* agglutinin [40]—also display binding affinity towards galactose/GalNAc and galactose/GalNAc containing polysaccharides. A characteristic, although not completely conserved, sequence feature of ricin B lectin domains is the presence of a glutamine-any residue-tryptophan motif (QxW) as internal repeats near the origin of the fourth β-strand of each subdomain (QxW)_3_ [41]. The tryptophan consistently forms part of the hydrophobic core [42], whereas the glutamine is hypothesised to be a putative carbohydrate binding residue [43]. Tpp80Aa1 does not have any fully conserved QxW motifs but does have conserved hydrophobic residues (either phenylalanine or tryptophan) in the ‘W’ position (Figure 3C and Figure S5). There is also a conserved aspartic acid in the second β-strand, and a ‘QQY’ repeat in the third β-strand of each domain, which have been proposed as putative carbohydrate binding sites in related structures (Figure 3C and Figure S5).

### 2.6. Tpp80Aa1 Binds Galactose-Containing Glycolipids and Lipids from Target Species

**Figure 4:**
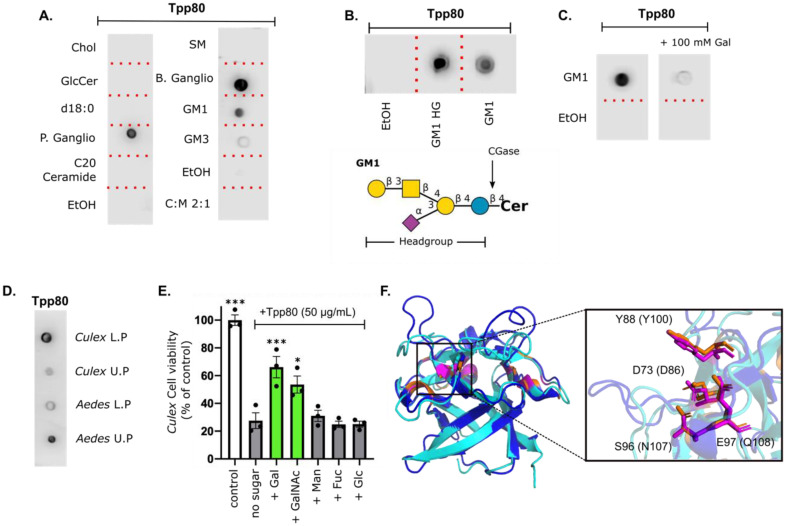
Tpp80Aa1 interacts with glycolipid moieties containing a terminal galactose residue. (**A**) Dot blots probed with biotinylated activated Tpp80Aa1 (20 μg/mL) show binding to porcine and bovine mixed gangliosides (P/B Ganglio), and ganglioside GM1 and GM3. No binding is observed to cholesterol (Chol), glucosylceramide (GlcCer), sphinganine (d18:0 Sph), C20 ceramide, or sphingomyelin (SM), and solvent only (C:M 2:1 or EtOH). (**B**) Tpp80Aa1 can bind the isolated sugar headgroup (HG) of GM1—CGase was utilised to cleave the sugar headgroup off GM1 as depicted in GM1 glycan (created using DrawGlycan 2.0 using standard sugar symbols). (**C**) Addition of galactose (100 mM) to the binding assay significantly reduces Tpp80Aa1 binding to GM1. (**D**) Tpp80Aa1 binds upper and lower phase lipid(s) isolated from *C. quinquefasciatus* and *Ae. aegypti* larvae. (**E**) Competition assay investigating the protective effects of sugars (15 mM) in the *C. quinquefasciatus* MRA-918 cell line. Galactose (Gal) and N-acetylgalactosamine (GalNAc) addition alongside Tpp80 reduces Tpp80 induced cytotoxicity (green bars). Mannose (Man), fucose (Fuc) and glucose (Glc) do not affect Tpp80-induced toxicity (grey bars). Statistical analysis was performed using GraphPad Prism (ver 8.2.0), using one-way ANOVAs comparing each group to the Tpp80 treated * *p* < 0.05, *** *p* < 0.01 (**F**) Alignment generated in PyMOL of Tpp80Aa1 (cyan) with Tpp78Aa1 (dark blue) with putative carbohydrate binding sites shown (Tpp78 = orange, Tpp80 = pink).

Given the presence of the ricin_B lectin domain, we investigated the ability of trypsin-activated Tpp80Aa1 to bind carbohydrate residues. Based on the structural similarity between the N-terminus of Tpp80Aa1 and other galactose-binding lectins, we utilised glycolipids as a tool to investigate the ability of Tpp80Aa1 to bind galactose. Lipid binding blots (Figure 4A) show Tpp80Aa1 can bind mixed ganglioside extracts (which contain GM1, GD1a, GD1b, GT1b), purified GM1, and purified GM3, but does not interact with glucosylceramide, C20 ceramide, sphingomyelin, phosphatidylcholine, cholesterol, ceramide phosphoethanolamine, or lysosphingomyelin. This indicates specific binding activity of Tpp80Aa1 to glycolipid structures with terminal β-D-galactose residues (GM1, GM3, GD1a, GD1b, GT1b) and no interaction with a terminal glucose (GlcCer) or the lipid moieties. In the case of GM3 binding, this also indicates that Tpp80Aa1 can bind a terminal galactose residue conjugated to a sialic acid residue. Indeed, Tpp80Aa1 can strongly bind the sugar headgroup of GM1 after it had been cleaved from the lipid fraction (Figure 4B) and adding galactose as a competitive inhibitor substantially reduced GM1 binding (Figure 4C). Collectively these experiments indicate the observed binding is via an interaction with the glycolipid headgroup.

To see if trypsin-activated Tpp80Aa1 was interacting with mosquito-derived lipids, we isolated lipids from the guts of *Culex* and *Aedes* mosquitoes into two chemical phases—the upper of which is hydrophilic and attracts glycolipids with more polar carbohydrate structures and the hydrophobic lower fraction which contains non-polar lipids which tend to have less complexity or no sugar structure [44,45]. We observed binding to both phases (Figure 4D), indicating Tpp80Aa1 can interact with mosquito-derived lipids. Binding to the upper phase suggests binding to polar glycolipids, presumably in part through galactose binding. Further work is required to validate if glycolipid interaction occurs in vivo: it is equally possible that Tpp80Aa1 is also capable of interacting with a galactose-containing glycoprotein.

### 2.7. Galactose Competition Reduces Tpp80Aa1 Toxicity in Mosquito Cell Lines

To investigate the biological relevance of galactose interaction, we performed sugar inhibition assays using the MRA-918 cell line from target species *C. quinquefasciatus* (Figure 4E). Addition of trypsin-activated Tpp80Aa1 rendered cells 28% viable compared to untreated controls, yet addition of Tpp80Aa1 alongside galactose or N-acetyl-galactosamine has a protective effect and resulted cells being 66% and 54% viable, respectively. Addition of mannose, fucose and glucose had no protective effect (Figure 4E). The addition of sugars alone had no effect on cell viability (Figure S6). These results show addition of external galactose or GalNAc significantly decreases Tpp80Aa1 cytotoxicity.

A structural alignment of the Tpp80Aa1 N-terminal lectin domain with the recently identified galactose-interacting Tpp78Aa1 (PDB: 7Y78) shows the trefoil domain adopts a highly similar conformation (RMSD 0.927 Å, Figure 4F), as was noted previously with an alignment between Tpp78Aa1 and ricin B [15]. Tpp78Aa1 has four conserved residues (D86, Y100, N107, and Q108) with ricin B (PDB: 3VT1) that are attributed to binding and recognition of galactose (D416, Y431, N438, and Q439). In Tpp80Aa1 we see a similar conservation (D73, Y88, S96, and E97) where the sidechains superimpose with those present in Tpp78Aa1, and we propose this as the putative galactose binding site of Tpp80Aa1 (Figure 4F). These conserved residues are present in each of the 3 subdomains within the β-trefoil of Tpp80Aa1 (Figure 3C), indicating multiple putative sugar binding sites.

## 3. Discussion

Tpp80Aa1 is an interesting novel candidate for mosquito control, having recently been shown to cause mortality in *Culex pipiens pallens* larvae [3], and its demonstrated target range has been expanded in this work to include *C. quinquefasciatus*, *Ae. aegypti*, and *An. gambiae*. Tpp80Aa1 may also be an option for circumventing resistance (such as in Tpp1/Tpp2 resistant *Culex*) or combining with other mosquitocidal proteins to lower the chance of resistance development. Compared to the other Tpp family members, Tpp80Aa1 is the only one capable of exerting mosquitocidal activity alone, although, in contrast to the other mosquitocidal Tpp binary toxins (Tpp1/Tpp2, Tpp49/Cry48) the potency appears to be lower, with approximately a 1000-fold higher concentration required for activity—although the variable nature of bioassays make this difficult to compare directly. Conceivably, the binary nature of these proteins facilitates a higher potency, with other Tpp proteins reported to act alone against insect targets also showing a lower potency: Tpp36 against western corn rootworm (147.3 µg/well) [46]; Tpp78Aa1 and Tpp78Ba1 against their rice planthopper targets (between approximately 6 and 16 µg/mL) [47,48]. Here, we demonstrate Tpp80Aa1 is localised and internalised in the same regions of the mosquito larval midgut epithelium as the Tpp1/Tpp2 complex [7]. Coadministration of Cyt1A protein has been observed previously to facilitate Tpp1 internalisation in resistant mosquito populations where Tpp2 no longer binds to the Cqm1 receptor [49]. The ability of Cyt1A to act as a surrogate receptor for Tpp80Aa1 in the same manner remains to be investigated.

The fact that Tpp80Aa1 acts alone makes it particularly appealing for manipulation to understand the mechanisms underlying insecticidal activity, and for engineering mutants to increase/alter toxicity—for which our resolved structure of Tpp80Aa1 provides a template. Tpp80Aa1 consists of a ricin B-type N-terminal trefoil domain and a C-terminal putative pore forming domain. Lectin domains are commonplace in domain 1 of Tpp proteins, and domain 3 of Cry toxins, suggesting a wider role for carbohydrate binding in pesticidal activity. This is indeed the case with some pesticidal proteins, as illustrated by glycosphingolipid receptors mediating Cry5B and Cry14A toxicity in nematodes [45], and N-acetylgalactosamine (GalNAc) forming a component of the Cry1Ac receptor(s) in some lepidopteran species [50,51]. In terms of the Tpp family, Tpp78 has recently been identified to interact with galactose, GalNAc, and lactose, and several sugars—including chitobiose, chitotriose, N-acetylmuramic acid and N-acetylneuraminic acid—can reduce Tpp1/Tpp2 activity in *Culex* cells [35], with arabinose and fucose also shown to reduce Tpp1 toxicity towards *Culex* larvae [52]. Within the Tpp80Aa1 β-trefoil, we recognise three putative carbohydrate binding domains—one in each of the α, β & γ subdomains, indicating the potential to interact with several–sugar molecules simultaneously. Further investigation will be required to confirm if these putative sites are facilitating the Tpp80Aa1 galactose interaction, and whether other carbohydrates—such as GalNAc—can also interact with Tpp80Aa1.

Lipids are known to play crucial roles in the mode of action of most protein toxins through promoting binding, endocytosis, and/or cytoplasmic translocation [53]. Examples include cholera toxin binding to GM1 [54], anthrax toxin to lipid microdomains [55], Shiga toxin to Gb3 [56], and lysenin binding to sphingomyelin [57]. Initial binding on the cell surface is hypothesised to initiate toxin oligomerisation—a critical step for facilitating conformational change, receptor recognition, and pore formation with β-PFTs. We suggest Tpp80Aa1 oligomerisation may occur through an interaction with galactose or GalNAc present on proteins or lipids at the cell surface. Indeed, addition of galactose/GalNAc to our cell bioassays has a protective effect on the cells, suggesting Tpp80Aa1 binding to free galactose/GalNAc is preventing it from interacting with its putative receptor(s). We observed binding to lipids isolated from target species, indicating that lipid binding occurs—although whether this facilitates toxin action is still to be investigated. As terminal galactose residues are not specific to glycolipids or glycoproteins of the mosquito midgut, it is highly likely other receptor(s) are present in the mosquito midgut to confer target species/ tissue specificity. However, a change in glycan binding profiles might be an indication of resistance development, as is exampled in previous studies investigating Cry5B toxicity in nematodes [45].

A two-stage binding process is hypothesised for many insecticidal toxins, with an initial low affinity interaction allowing the flexibility required for reorganisation into an oligomeric state prior to receptor interaction [58,59]. A well characterised example is the initial low-affinity interaction of Cry1Ac to GalNAc followed by secondary high-affinity binding to a glycoprotein receptor [60,61]. For the Tpp1/Tpp2 binary complex, receptor-mediated endocytosis appears to be a key component of pore formation, as demonstrated by the formation of cationic ion channels in—normally nontarget—MDCK cell lines engineered to express the relevant Cqm1 receptor [17]. Furthermore, the Cqm1 receptor is localised to lipid microdomains enriched in glycosphingolipids which could be playing an important role in initial oligomerization. Precisely how the pore-forming domain in Tpp proteins inserts into the membrane is unknown.

Future experiments to confirm pore forming activity and discover the Tpp80Aa1 receptor will be key to understanding its mechanism of action. Here, we pinpoint both putative carbohydrate-binding residues and residues hypothesised to initiate membrane-insertion and pore formation. This work can facilitate future studies exploring the mechanism of action, enhancing Tpp80Aa1 activity, and developing Tpp80Aa1 for potential use in the field.

## 4. Materials and Methods

### 4.1. Tpp80Aa1 Expression and Purification

A synthetic clone of the *tpp80Aa1* gene was produced in the pET30a plasmid to express a Tpp80Aa1 protein with a short N-terminal extension including a hexa-histidine tag (by inserting the entire *tpp80Aa1* reading frame, downstream of the *Bam*HI site in the vector). This plasmid was introduced into BL21 *E. coli* cells and cultured in 2× YT medium containing kanamycin. Once the OD_600_ reached ~0.6, protein expression was induced with 0.5 mM IPTG for 18 h with shaking at 25 °C (200 rpm). Bacterial cultures were collected (7000× *g*, 4 °C, 15 min) and lysed via two freeze–thaw cycles (−80 °C/37 °C) and sonication (10 × 10 s, with 20 s intervals, on ice). The lysate was clarified by centrifugation (23,000× *g*, 4 °C, 30 min) and then filtered through a 0.45 μm filter and proteins were purified using standard immobilised metal affinity chromatography (Protino Ni-TED, Macherey-Nagel, Düren, Germany). Samples were concentrated and imidazole removed via buffer exchanging the sample by 4 rounds of dilution/concentration in a 10 kDa cut-off centrifugal filter unit (Amicon Ultra-15, MilliporeSigma, Burlington, USA using 50 mM TrisHCl pH 7.4 supplemented with a protease inhibitor cocktail (cOmplete Protease Inhibitor Cocktail, Roche, Basel, Switzerland). Purified proteins were examined by SDS-PAGE. For ultra-pure samples, eluted samples were subjected to size-exclusion chromatography (SEC; Figure S1). Where trypsinised protein was required (all presented dot blots and cell assays), immobilised TPCK treated trypsin resin (20233, Thermo Scientific, Waltham, MA, USA) was added to the sample and incubated in a shaker at 37 °C for 16 h, followed by centrifugation to remove the trypsin resin prior to use. Liquid chromatography mass spectrometry (LCMS) was used to quantify molecular weight, and N-terminal sequencing by Edman degradation was performed by Alta Bioscience (Birmingham, UK).

### 4.2. Bioassays (Insects & Cells)

Bioassays were carried out against a range of mosquito larvae (*C. quinquefasciatus*, *Ae. aegypti*, and *An. gambiae*) and insect cell lines derived from *C. quinquefasciatus* (MRA-918), *Ae. aegypti* (C6/36) and *An. gambiae* (Ag55). MRA-918 cells were kindly gifted by Dr. Mario Soberón (Mexico City, Mexico) and c6/36 and Ag55 cells from Dr. Claire Donald (Glasgow, UK). For insect larvae, 10–15 third-instar larvae were placed in 5 mL of dH_2_O and maintained in a humidified room at 24 °C. Mortality was assessed by counting live larvae at 24 h after the addition of purified toxin, or the equivalent amount of the relevant buffer (50 mM TrisHCl, pH 7.5). For all larval bioassays, non-trypsinised protein was used. The concentration giving 50% mortality (LC_50_) was calculated using GraphPad prism for Mac OS (Ver 8.2.0) plotting log(concentration of toxin) against % survival rate.

Insect cell lines were maintained at 27 °C in Schneider’s Insect Medium supplemented with 10% FBS. For cellular bioassays, cells were plated at 10,000/well of a 96-well plate in 150 μL of medium until ~70% confluent. Cell viability was investigated using resazurin, as described previously. Trypsinised Tpp80Aa1 was used in cellular bioassays. For the sugar competition assay, sugars (glucose, galactose, mannose, fucose, N-acetylgalactosamine) were dissolved into the cell culture medium at a final concentration of 15 mM, alongside activated Tpp80 and sugar-only controls. GraphPad Prism for Mac OS (Ver 8.2.0), using one-way ANOVAs followed by Dunnett’s multiple comparisons test was used to compare individual treatment groups back to the control. Data are presented as mean ± standard deviation.

### 4.3. Tpp80Aa1 Labelling

Fluorescent labelling of Tpp80Aa1 (non-activated) for midgut imaging was performed using the Alexa Fluor^TM^ 488/555 Protein Labelling Kit (A10235, Invitrogen, Waltham, MA, USA), as per the manufacturer’s instructions. Briefly, Tpp80Aa1 was diluted to 2 mg/mL in dPBS in a final volume of 0.5 mL, to this 50 μL of 1 M sodium bicarbonate buffer (pH 8.3) was added. Tpp80Aa1 solution was added to the dye-containing vial and stirred at RT for 1 h. Purification of labelled protein was achieved using the Zeba Dye Spin Columns provided. Labelled protein (Tpp80Aa1-555) was stored at 4 °C protected from light.

### 4.4. In Vivo Midgut Imaging

Labelled Tpp80Aa1-555 protein was added to 1.5 mL water at a final concentration of 50 μg/mL containing 4 mosquito larvae (third instar). After 45 min, larvae were put in fresh water (containing no Tpp80Aa1-555) and left for a further 30 min before gut dissection in PBS. To label cell nuclei, extracted guts were added to PBS containing 1 μg/mL Hoechst 33342 and gently rocked at RT for 30 min. Samples were mounted in 1 mm glass capillary tubes (10490413, Fisher Scientific, Waltham, MA, USA) with 1% low melting point agarose (16520050, ThermoFisher, Waltham, MA, USA). For imaging, we used a Zeiss Lightsheet Z.1 (Oberkochen, Germany) with a 405 nm (20 mW) and 561 nm laser (20 mW) with either a 10×/0.5 W Plan Apo or a 20×/1.0 Plan Apo (water immersion) objective.

### 4.5. Crystallisation

For crystallisation trials, Tpp80Aa1 was concentrated to 8 mg/mL in 50 mM TrisHCl pH 8.0. Crystallisation screening was performed in 96-well plates (3 Lens Crystallisation plate, SWISSCI, Zug, Switzerland) using a commercially available crystal screen, PACT Premier HT-96 screen (MD1-36, Molecular Dimensions, Rotherham, UK). Plates were set up using a Mosquito Crystallisation robot (SPT Labteck, Melbourn, UK), with 200 nL Tpp80Aa1 added to 200 nL screen solution. Very small crystals grew in several wells, the most promising single crystals appeared in F11 condition (0.2 M sodium citrate, 0.1M Bis Tris propane, 20% *w*/*v* PEG 3350, pH 6.5) and were used for seeding preparation. For making seed stock, 20 μL of F11 reservoir buffer was added to crystal drops, crystals were then crushed with a glass crystal crusher and transferred to an Eppendorf containing a PTFE seed bead for brief centrifugation. Crystal seeds were diluted 1:10 with well solution and added to the same 96-well screen (200 nL seed dilution, 200 nL protein, 200 nL buffer). Seeding produced multiple hits, which were harvested 2-weeks post seeding and flash-frozen in liquid nitrogen. H11 condition (0.2 M sodium citrate, 0.1 M Bis Tris propane, 20% *w/v* PEG3350, pH 8.5) produced the 1.8 Å Tpp80Aa1 dataset.

### 4.6. Data Collection and Structure Determination

Data were collected at Diamond Light Source (Harwell, UK) at beamline I03. Images were processed with the DIALS package and amplitudes estimated with TRUNCATE, in the CCP4 package [62]. The structure of Tpp80Aa1 was determined using molecular replacement in PHASER using the CCP4i2 software (ver 7.1.012). The starting search model was a synthetic construct, ThreeFoil (PDB entry 3PG0), and the C-terminus (aa 159–366) of Tpp1 (formerly BinA, PDB entry 5FOY). This was followed by successful model building of a partial model with Buccaneer. The resulting model and maps were inspected manually via Coot [63], followed by iterative rounds of real-space refinement and model building cycles using Coot and REFMAC5 [64], respectively. Data collection and processing statistics, and refinement statistics are summarised in Table S1.

### 4.7. Structural Analysis

Comparing Tpp80Aa1 structural similarity to other proteins in the Protein Data Bank was performed using the DALI server and a heuristic PDB search [65]. Interface analysis was performed using the PDBe PISA web server [66].

### 4.8. Lipid Extractions

Lipids were purified from larvae into two chemical phases using Svennerholm partitioning. Larvae were starved for 24 h prior to lipid extraction. Second/third instar larvae (0.5 g total mass) were rinsed three times in dH_2_O, then flash frozen in liquid nitrogen and thawed at RT three times. Pellets were sonicated and lipid extraction was performed at 37 °C for 2 h with agitation in a mixture of chloroform, methanol, and water with a final ratio of 4:8:3. Samples were centrifuged at 1400× *g* for 5 min to split into upper (hydrophilic, attracts more polar lipids) and lower (hydrophobic, attracts generally simpler nonpolar lipids) phases. Silica-based hydrophobic cartridges (WAT036810, Sep-Pak tC18, Wilmslow, UK) were used to purify and concentrate upper phase glycolipids. All samples were dried under N_2_ at 40 °C and resuspended in 50 μL methanol (upper phase) and 200 μL 1:1 chloroform to methanol (lower phase) for use in dot blots. Thin layer chromatography was used to check successful lipid extraction (not shown).

### 4.9. Lipid Dot Blots

All dot blots were performed using a PVDF 0.2 μm pore membrane (ISEQ00010 Immobilon^®^-PSQ PVDF, Millipore, Burlington, MA, USA). Lipid standards were added to the blot at a concentration of 2 μg in a volume no larger than 4 μL, or for larvae-extracted lipids, 2 μL of the final suspension. Blots were left to dry and then blocked with tris buffered saline (TBS) containing 5% BSA for 1 h at room temperature (RT). Trypsin-activated Tpp80Aa1 was added at 20 μg/mL in TBS containing 1% BSA (TBS-1%) and left agitating overnight at 4 °C. Blots were washed in TBS-1% for 10 min at RT, and then probed with an anti-polyHistidine-Peroxidase antibody (A7058, Sigma-Aldrich, St. Louis, MO, USA) for 1 h at RT with agitation. Blots were washed in TBS-1% and visualised using a LI-COR C-Digit chemiluminescence Western blot scanner and a WESTAR ECL-Sun HRP detection kit, (K1-0052, geneflow, Lichfield, UK). For activated toxin, the same process was used but with biotinylated Tpp80Aa1 and an ABC-HRP kit (PK-6100, vector laboratories, Burlingame, CA, USA). The lipids used in this manuscript were; porcine brain total ganglioside extract (860053P Avanti^®^ Polar Lipids, 2 mg/mL in chloroform:ethanol 2:1), ganglioside GM3 (GM3, 860058P Avanti^®^ Polar Lipids, 2 mg/mL in ethanol), glucosylceramide (GlcCer, 131304P Avanti^®^ Polar Lipids, 2 mg/mL in ethanol), ganglioside GM1 (GM1, 860065P Avanti^®^ Polar Lipids, 2 mg/mL in ethanol), mixed bovine gangliosides (1065 Matreya LLC, 10 mg/mL in chloroform:ethanol 2:1), C20 ceramide (860520P Avanti^®^ Polar Lipids, 10 mg/ mL in ethanol), sphingomyelin (860062 Avanti^®^ Polar Lipids, 10 mg/mL in chloroform), cholesterol (700000P Avanti^®^ Polar Lipids, 2 mg/mL in ethanol), and, sphinganine d18:0 (860498P Avanti^®^ Polar Lipids, 2 mg/mL in ethanol). For the galactose competition assay, GM1 was added to the blot as described above, and 100 mM galactose was added into the Tpp80Aa1-containing TBS solution. Ceramide glycanase (LZ-CER-HM-KIT, LudgerZyme, Oxfordshire, UK) was used to remove the sugar headgroup from GM1, as per instructions from the supplier. Briefly, 10 μL of enzyme was added per 2 nmol of GM1 alongside 10 μL of reaction buffer and 16 μL of dH_2_O, and the resulting solution was incubated at 37 °C for 24 h. To purify the glycan from the mixture, LudgerClean S cartridges (LC-S-A6, LudgerZyme, Oxfordshire, UK) were used as per the supplier’s instructions. Eluted glycans were dried and resuspended in 50 μL ethanol prior to dot blot assay.

## Data Availability

The data presented in this study are openly available in the Protein Data Bank at www.rcsb.org with accession code 8BAD.

## Supplementary Materials

The following supporting information can be downloaded at: https://www.mdpi.com/article/10.3390/toxins14120863/s1, Figure S1: Tpp80Aa1 expression, crystallisation, and N-terminal sequencing; Figure S2: Electron density map and model of the Tpp80Aa1 structure; Figure S3: Superposition and interfaces of the Tpp80Aa1 monomers; Figure S4: Conserved putative insertion loop contacts in Tpp family members; Figure S5: Multiple sequence alignment of QxW motifs in Tpp80Aa1 and structurally similar lectin domains; Figure S6. Sugar addition had no effect on MRA-918 cell viability. Galactose (Gal), N-acetylgalactose (GalNAc), mannose (Man), fucose (Fuc) or glucose (Glu) were added to C. quinquefasciatus derived cells (MRA-918) at a final concentration of 15 mM. Twenty-four hours post addition, no significant impact was observed on cell viability, as quantified by resazurin assay (*p* > 0.05, one-way ANOVA compared to control); Table S1: Data collection and refinement statistics for Tpp80Aa1; Table S2: Interfaces in the Tpp80Aa1 crystal structure, as calculated by PDBePISA; Table S3: Top 20 proteins with structural similarity to Tpp80Aa1, as identified by the DALI server. References [67,68,69,70,71,72,73,74,75,76,77] are cited in the supplementary materials.
