# Supplementary material for: The Crystal Structure of Bacillus thuringiensis Tpp80Aa1 and Its Interaction with Galactose-Containing Glycolipids
Source: Toxins (Basel). 2022 Dec 8;14(12):863. doi: 10.3390/toxins14120863 (PMC9784298; doi:10.3390/toxins14120863)
Supplement: Supplementary file 1 [file toxins-14-00863-s001.zip › toxins-2040583-supplementary.pdf]

Article

The Crystal Structure of *Bacillus thuringiensis* Tpp80Aa1 and Its Interaction with Galactose-Containing Glycolipids

Hannah L. Best, Lainey J. Williamson, Magdalena Lipka-Lloyd, Helen Waller-Evans, Emyr Lloyd-Evans, Pierre J. Rizkallah and Colin Berry

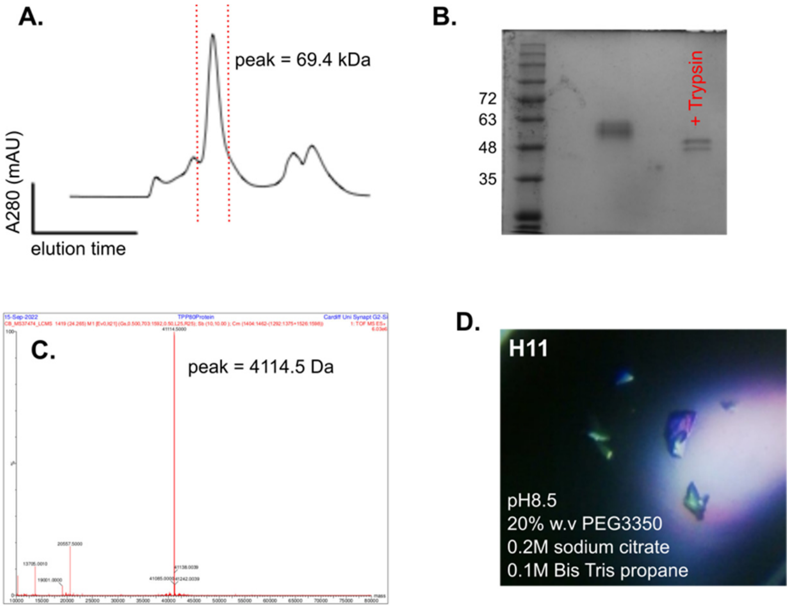

Figure S1. Tpp80Aa1 expression, crystallisation, and N-terminal sequencing. (A) Tpp80Aa1 was purified using size exclusion chromatography (SEC) and fractions from the main peak (69.4 kDa) concentrated prior to crystal tray setup. (**B**) Purified Tpp80Aa1 with and without proteolytic activation by trypsin. (**C**) LCMS shows the size of the proteolytically cleaved Tpp80 to be 41114.5 Da, consistent with a monomeric form. (**D**) Crystals which produced the Tpp80Aa1 structural dataset.

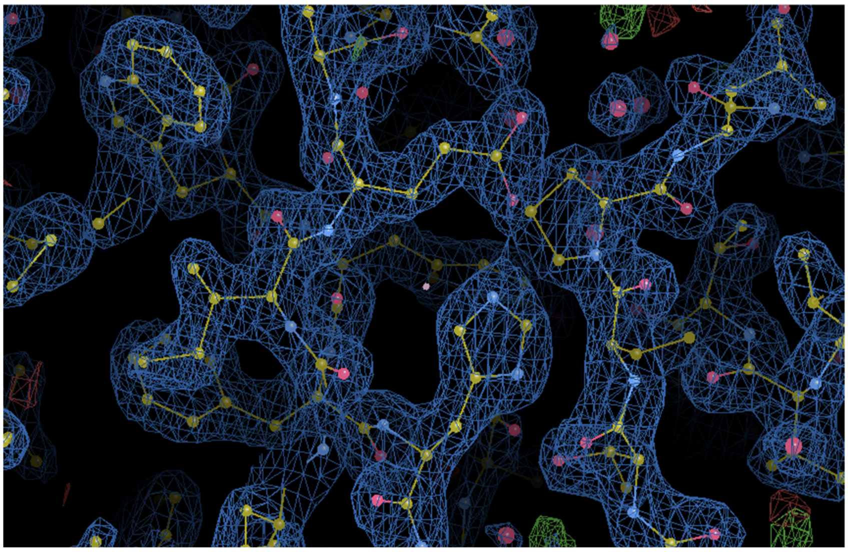

Figure S2. Electron density map and model of the Tpp80Aa1 structure. A segment through the centre of a Tpp80Aa1 monomer is shown in COOT. Protein backbone is coloured yellow, nitrogen atoms are blue, and oxygen atoms are pink. .

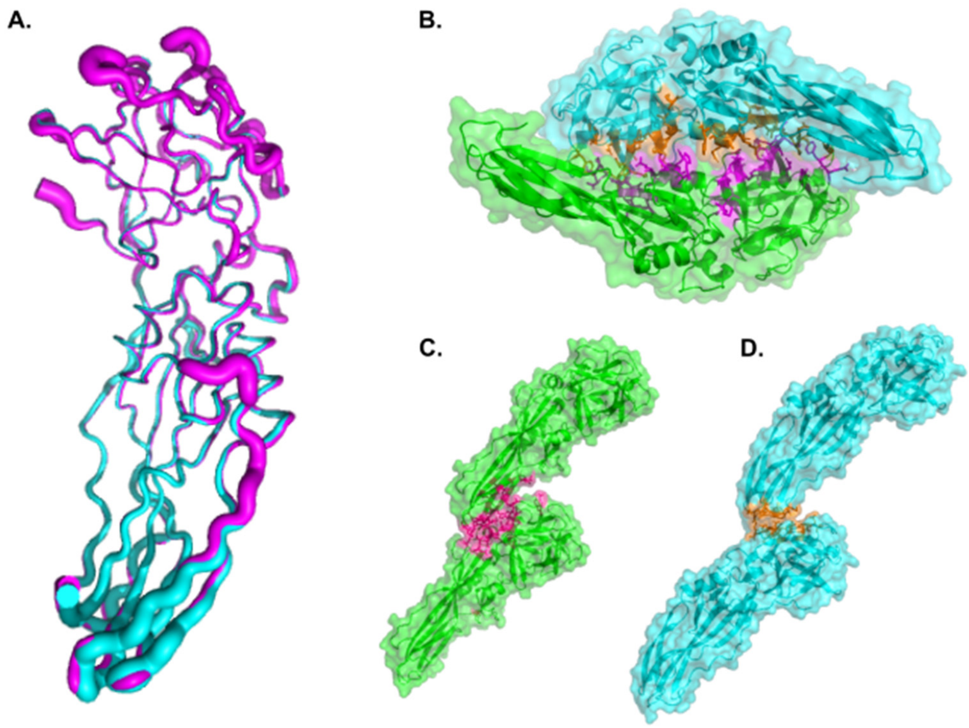

Figure S3. Superposition and interfaces of the Tpp80Aa1 monomers. (**A**) Superposition of Tpp80Aa1 monomers by PyMOL - represented by B-factor putty to show the magnitude of displacement of the atoms from their central positions - indicates the two copies to be highly similar, with an RMSD of 0.795 Å. (**B**) Dimer interface highlighting the that the regions amenable to proteolytic cleavage (N-terminal region) could be partially buried at a crystal interface. Monomer A is shown in green with interfacing residues in pink, monomer B in cyan with interfacing residues in orange. (**C**) and (**D**) highlight where the extreme C-terminus of A and B monomers form part of a crystal contact with symmetry mates in other dimers within the crystal.

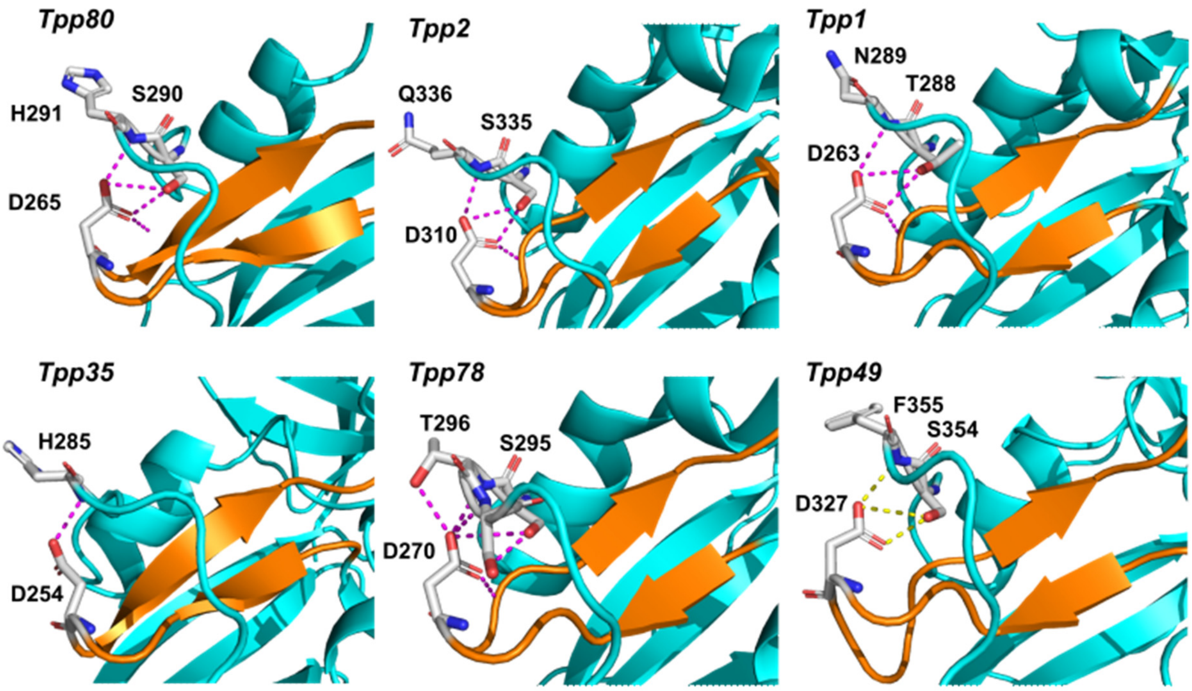

Figure S4. Conserved putative insertion loop contacts in Tpp family members. All Tpp family members have a putative insertion β-hairpin (orange) tucked under a loop (cyan). Sequence alignment of the putative insertion loop from Tpp family members showed the presence of a conserved aspartic acid (D) residue at the tip of the loop. This conserved residue forms polar contacts with residues in the loop above it (pink dotted lines).

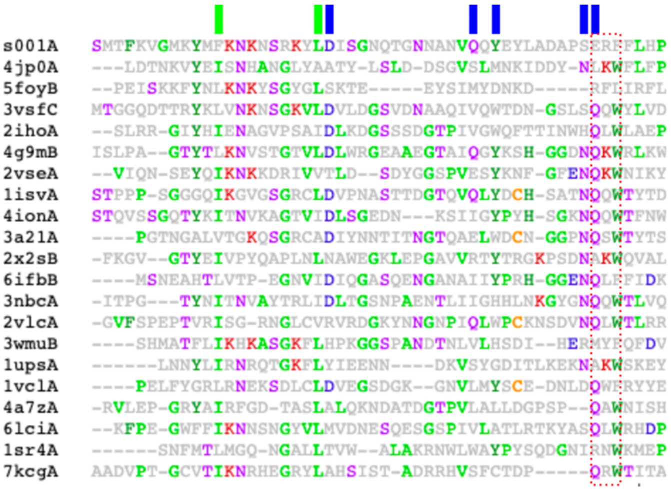

Figure S5. Multiple sequence alignment of QxW motifs in Tpp80Aa1 and structurally similar lectin domains. QxW lectin domain internal repeats are a not completely conserved, characteristic of ricin-like lectin domains. Alignment of the α subdomain of the lectin domain from Tpp80Aa1 (s001A, top) with structurally similar proteins (identified by PDB number) that also contain lectin domains, highlights sequence conservation. Putative carbohydrate binding site residues are highlighted by a blue box above the alignment. The residues highlighted by a green box are part of the conserved hydrophobic core, and the QxW motif is highlighted by a red dotted box. Alignment was generated by PDBePISA, the most conserved residue at each position is coloured, purple = polar, blue = negatively charged sidechain, dark green = aromatic, red = positively charged side chain, lime green = hydrophobic side chain.

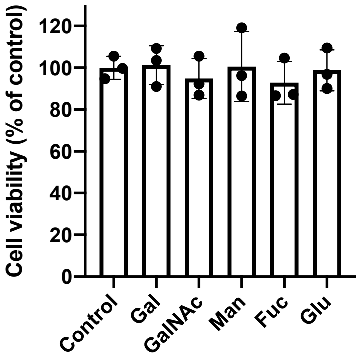

Figure S6. Sugar addition had no effect on MRA-918 cell viability. Galactose (Gal), N-acetylgalactose (GalNAc), mannose (Man), fucose (Fuc) or glucose (Glu) were added to *C. quinquefasciatus* derived cells (MRA-918) at a final concentration of 15 mM. Twenty-four hours post addition, no significant impact was observed on cell viability, as quantified by resazurin assay ($p > 0.05$, one-way ANOVA compared to control).

Table S1. Data collection and refinement statistics for Tpp80Aa1.

**PDB Entry**	**8BAD**
**Data Collection***	
Diamond Beamline	I03
Date	2022-05-27
Wavelength	0.97628
**Crystal Data (figures in brackets refer to outer resolution shell)**	
Crystallisation Conditions	0.02M sodium/potassium phosphate<br>0.1M Bis-tris propane<br>20% w/v PEG3350<br>pH 7.5
a,b,c (Å)	133.827, 63.853, 107.292
, , °	90.0, 120.40, 90.0
Space group	C 1 2 1
Resolution (Å)	1.81 – 57.73
Outer shell	1.81 – 1.84
R-merge (%)	10.8 (224.3)
R-pim (%)	4.8 (102.0)
R-meas (%)	12.8 (265.4)
CC1/2	0.998 (0.261)
I / σ(I)	11.8 (0.6)
Completeness (%)	100 (100)
Multiplicity	7.0 (6.7)
Total Measurements	498,808 (23,361)
Unique Reflections	71,433 (3,495)
Wilson B-factor (Å^2)	25.5
**Refinement Statistics**	
Refined atoms	6,120
Protein atoms	5,680
Non-protein atoms	91
Water molecules	349
R-work reflections	67,839
R-free reflections	3,513
R-work/R-free (%)	17.9 / 21.0
**rms deviations (target in brackets)**	
Bond lengths (Å)	0.011 (0.013)
Bond Angles (°)	1.338 (1.656)
[1]Coordinate error	0.125
Mean B value (Å^2)	36.9
**Ramachandran Statistics (PDB Validation)**	
Favoured/allowed/Outliers %	
0	

Table S2. Interfaces in the Tpp80Aa1 crystal structure, as calculated by PDBePISA.

	Monomer 1			Monomer 2			Interface					
	**Range**	**$^iN_{at}$**	**$^iN_{res}$**	**Range**	**$^iN_{at}$**	**$^iN_{res}$**	**Interface area Å²**	**Δ^iG kcal/mol**	**Δ^iG P-value**	**N_{HB}**	**N_{SB}**	**N_{DS}**
**1**	B	127	34	A	135	35	1010.9	-1.8	0.568	12	0	0
**2**	B	71	22	B	71	22	658.7	1.2	0.734	10	8	0
**3**	A	65	21	A	64	20	589.9	2.1	0.747	8	5	0
**4**	A	53	17	A	35	10	362.1	1.3	0.681	2	0	0
**5**	B	37	10	B	42	16	361.0	-3.3	0.205	2	0	0
**6**	A	13	5	B	21	7	164.3	-0.4	0.280	2	1	0
**7**	B	18	5	B	18	5	161.7	1.5	0.798	0	0	0
**8**	A	14	6	B	16	6	159.5	0.3	0.629	2	0	0
**9**	B	2	2	B	3	2	21.2	0.4	0.786	0	0	0
**10**	A	3	2	A	3	2	18.7	0.3	0.723	0	0	0
**11**	B	1	1	B	1	1	2.7	0.0	0.541	0	0	0

Range indicates chain ID, **$^iN_{at}$** indicates the number of interfacing atoms in the corresponding structure, ***$^iN_{res}$*** indicates the number of interfacing residues in the corresponding structure, ***Surface Å²*** is the total solvent accessible surface area in square Ångstroms, ***Interface area*** in Å², calculated as difference in total accessible surface areas of isolated and interfacing structures divided by two, ***Δ^iG*** indicates the solvation free energy gain upon formation of the interface, in kcal/M, ***Δ^iG P-value*** indicates the P-value of the observed solvation free energy gain, ***N_{HB}*** indicates the number of potential hydrogen bonds across the interface, ***N_{SB}*** indicates the number of potential salt bridges across the interface, and ***N_{DS}*** indicates the number of potential disulphide bonds across the interface. .

Table S3. Top 20 proteins with structural similarity to Tpp80Aa1, as identified by the DALI server.

**Name**	**Carbohy-drateLigand(s)**	**PDB ID**	**Z-score**	**RMSD (Å)**	**Sequence iden-tity (%)**
Tpp35Ab1 *(Bacillus thuringiensis)*	-	4JP0-A	33.2	2.9	22
Tpp2Aa2 *(Lysinibacillus sphaericus)*	-	5FOY-B	31.8	3.9	22
1,3Gal4,3A *(Clostridium thermocellum)*	Glycerol	3VSF-C	27.5	1.0	24
MOA *(Marasmius oreades)*	Gal(1,3)Gal(1,4)GlcNAc [67]	2IHO-A	24.3	7.5	12
Agglutinin (*Rhizoctonia solani)*	GalNAc [68]	4G9N	23.5	1.3	17
Mtx1Aa1 holoprotein *(Lysinibacillus sphaericus)*	-	2VSE-A	23.0	14.4	17
E-86 xylanase *(Streptomyces olivaceoviridis)*	xylose, xylobiose, xylotriose, Glc, Gal, Lac [69]	1ISV-A	22.3	1.5	22
MPL ricin B-like lectin *(Macrolepiota procera)*	Glycerol	4ION-A	22.1	1.7	16
β-L-Arabinopyranosidase *(Streptomyces avermitilis)*	L-arabinose, Gal [70]	3A21-A	22.0	1.5	15
Agglutinin *(Sclerotinia scelrotiorum)*	Gal, GalNAc [71]	2X2S-B	21.7	1.7	16
β -trefoil lectin *(Entamoeba histolytica)*	Rhamnose, Gal, Gal-linked [72]	6IFB-B	21.1	2.4	20
CNL ricin B-like lectin *(Clitocybe nebularis)*	GalNAcβ1-4GlcNAc, LacdiNAc, Lac, LacdiNAc [73]	3NBC-A	20.7	1.8	15
Natural Cinnamomin hydrolase *(Cinnamomum camphora)*		2VLC-A	19.9	1.7	12
Mytilec *(Mytilus galloprovincialis)*	melibiose, Gb3, Gal, GalNAc [74]	3WMU-B	19.7	1.9	16
Glycosyl hydrolase *(Clostridium perfringens)*		1UPS-A	19.3	1.9	15
Hemolytic lectin CEL-III *(Cucumaria enchinata)*	Gal [75]	1VCL-A	19.2	2.9	21
Aldos-2-ulose dehydratase (*Phanerodontia chrysosporium)*	Glucosone, 1,5-D-anhydrofructose [76]	4A7Z-A	18.9	2.3	9
mdaA-1 *(Mucor circinelloides)*		6LCI-A	18.3	1.9	16
Cytolethal distending toxin *(Haemophilus ducreyi)*		1SR4-A	18.2	2.6	13
Salivary protein *(Culex quinquefasciatus)*	Gal, GalNAc, Gb4 [77]	7KCG-A	18.1	2.1	14
